# Accumulation and transport of microbial-size particles in a pressure protected model burn unit: CFD simulations and experimental evidence

**DOI:** 10.1186/1471-2334-11-58

**Published:** 2011-03-03

**Authors:** Christian Beauchêne, Nicolas Laudinet, Firas Choukri, Jean-Luc Rousset, Sofiane Benhamadouche, Juliette Larbre, Marc Chaouat, Marc Benbunan, Maurice Mimoun, Jean-Patrick Lajonchère, Vance Bergeron, Francis Derouin

**Affiliations:** 1 Electricité De France Research and Development, 6 quai Watier 78400 Chatou, France; 2 Airinspace SAS, Montigny, France; 3 Laboratory of Parasitology-Mycology, Saint-Louis hospital, Assistance Publique-Hôpitaux de Paris, and University Paris, Diderot, France; 4 Burn Centre, Department of Reconstructive/Plastic Surgery, Rothschild Hospital, Paris, France; 5 Cell Therapy Unit, Saint-Louis hospital, Assistance Publique-Hôpitaux de Paris, France; 6 Direction, Hôpital Saint-Louis, Assistance Publique-Hôpitaux de Paris, France; 7 CNRS UMR, Ecole Normale Supérieure de Lyon, 46 allée d'Italie, 69007, Lyon, France

## Abstract

**Background:**

Controlling airborne contamination is of major importance in burn units because of the high susceptibility of burned patients to infections and the unique environmental conditions that can accentuate the infection risk. In particular the required elevated temperatures in the patient room can create thermal convection flows which can transport airborne contaminates throughout the unit. In order to estimate this risk and optimize the design of an intensive care room intended to host severely burned patients, we have relied on a computational fluid dynamic methodology (CFD).

**Methods:**

The study was carried out in 4 steps: i) patient room design, ii) CFD simulations of patient room design to model air flows throughout the patient room, adjacent anterooms and the corridor, iii) construction of a prototype room and subsequent experimental studies to characterize its performance iv) qualitative comparison of the tendencies between CFD prediction and experimental results. The Electricité De France (EDF) open-source software *Code_Saturne*^® ^(http://www.code-saturne.org) was used and CFD simulations were conducted with an hexahedral mesh containing about 300 000 computational cells. The computational domain included the treatment room and two anterooms including equipment, staff and patient. Experiments with inert aerosol particles followed by time-resolved particle counting were conducted in the prototype room for comparison with the CFD observations.

**Results:**

We found that thermal convection can create contaminated zones near the ceiling of the room, which can subsequently lead to contaminate transfer in adjacent rooms. Experimental confirmation of these phenomena agreed well with CFD predictions and showed that particles greater than one micron (i.e. bacterial or fungal spore sizes) can be influenced by these thermally induced flows. When the temperature difference between rooms was 7°C, a significant contamination transfer was observed to enter into the positive pressure room when the access door was opened, while 2°C had little effect. Based on these findings the constructed burn unit was outfitted with supplemental air exhaust ducts over the doors to compensate for the thermal convective flows.

**Conclusions:**

CFD simulations proved to be a particularly useful tool for the design and optimization of a burn unit treatment room. Our results, which have been confirmed qualitatively by experimental investigation, stressed that airborne transfer of microbial size particles via thermal convection flows are able to bypass the protective overpressure in the patient room, which can represent a potential risk of cross contamination between rooms in protected environments.

## Background

Infections in burn patients are a major cause of morbidity and mortality due to cumulative risk factors such as the burn injury itself, the immunodeficiency related to extended burn wounds, aggressive therapy and prolonged hospitalization. Microorganisms that cause these infections include bacteria, fungi and viruses [1,2] and are commonly found in the patient's own endogenous flora, but can also originate from exogenous sources and from health care personnel. Noteworthy are *Acitenobacter, Pseudomonas *and resistant *Staphyloccocus aureus *which have been responsible for outbreaks in burn units [3,4]. Modes of transmission to the patient include contact, droplet and airborne spread [1,4]. Moreover, environmental conditions in these units call for elevated temperatures (>30°C) and humidity levels (>50% RH) which could facilitate microbial growth and environmental contamination.

Although the incidence of burn wound infections has declined in recent years, infection rates remain high in patients with burns that exceed 30% of the total body surface area [4]. Preventing direct transmission of infection in burn patients relies on strict application of disinfection and sterilization guidelines. The objective is to both prevent any microbial entry into the room, and simultaneously avoid any microbial diffusion from exiting and reaching adjacent rooms. This is particularly important for burn patients because of the high exposed surface area they have in contact with room air and their high propensity to endogenous acquired infections.

Infection risk is also influenced by the design of burn unit rooms, [5] and it has been shown that the use of single bed isolation [6,7], pre-emptive barrier precautions [8] and laminar airflow isolation [9] can be effective protection measures to prevent cross transmission and outbreaks [5]. However, basic knowledge of room design parameters is lacking and must take into account the unique environmental factors that these locations require (*e.g. *elevated temperature and humidity). In particular, thermo-convective air flows that can transport airborne contaminates from adjacent rooms and throughout the unit need to be considered. Thus, we have used Computational Fluid Dynamic (CFD), which incorporate thermal sources and the opening and closing of room access doors, as a tool for optimizing the design of an intensive care room for severely burned patients [10]. Based on these simulations, a prototype room was constructed and experimental control measurements were carried out and used for validation and direct comparison with the CFD predictions.

## Methods

The initial room design was made using knowledge acquired from the medical staff and hospital engineering department, taking into account that such rooms will be used for intensive care, complex dressing, showering and surgical treatment of burn patients, and that the patient's stay can last from up to 2 weeks to several months for the most severe cases.

**Figure 1:**
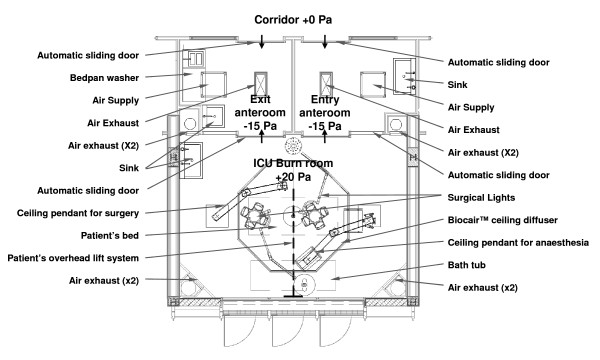
**Patient room and anteroom layout**. Top view of intensive care room for severely burned patients (size L5.95 × W4.3 × H2.9 m) and anterooms layout.

The basic room layout that emerged and was subsequently used for CFD simulations included a central large room under positive pressure (+20 Pa) for patient treatment, with two anterooms for entry and exit, both maintained at negative pressure (-15 Pa) relative to the adjacent corridor (0 Pa) (Figure 1). This overall configuration is meant to always channel air into the anterooms when transitioning between the patient room or the corridor, and corresponds to air distribution patterns in accordance with recommendations from the French standard NF S90351 for very high risk areas. Inlet air originates from a central octagonal ceiling plenum that has a diffusion array of 9 m^2 ^and 70 cm rigid Plexiglas curtains around its perimeter to channel air downward into the room from the ceiling. Prescribed airflow velocities to the treatment room were targeted at 0.25 m/s, which resulted in 90 air changes per hour (ACH) in the room (8100 m^3^/h). Inlet air temperature was 32°C. Two exhaust ducts are located in each corner of the room at 0.15 and 1.4 meters above the floor. The anterooms are provided with filtered air at 25 ACH (648 m^3^/hr), and maintained at 25°C, with sliding access doors leading to the treatment room and corridor. Air exhaust grilles are located on the ceiling near the center of each anteroom and air in the corridor was kept between 25-27°C.

### Computational Fluid Dynamic simulations (CFD)

CFD analysis of the initial room design was performed using the Electricité De France (EDF) software *Code_Saturne*^® ^[11]. The software is based on a Eulerian unstructured co-located finite volume approach that solves incompressible Navier-Stokes equations for meshes with cells of any shape (tetrahedral, hexahedral, prismatic, pyramidal, polyhedral...). The meshes can be grid structured and non conforming. The predictor-corrector method is used for pressure velocity coupling (SIMPLEC algorithm). The transport equation that takes into account the temperature is then solved with an updated velocity. The code does not use by default a Boussinesq approximation but a variable density with a conservation of the mass flow (the time derivative of the density is neglected in the continuity equation at low Mach numbers).

The turbulence model used is a standard k-ε model. The buoyancy forces, which depend only on the temperature, are thus taken into account as an explicit source term in the momentum equations via the turbulent kinetic energy and turbulent dissipation equations. A second moment closure that takes into account anisotropy effects of turbulence and in particular of buoyancy production would have been more appropriate but has not been used herein due to a lack of CPU time. Moreover, the present two equations model seems to be sufficient to obtain satisfactory qualitative results (see comparison with experimental results). Due to the relative simplicity of the turbulence model used for the flow dynamics, the turbulent heat fluxes are modelled using a Simple Gradient Diffusion Hypothesis (SGDH). The turbulent Prandtl number is taken equal to 1. Table 1 summarizes the boundary conditions used in the present work. These conditions are standard in CFD computations and no particular treatment has been introduced in the present study.

**Table 1:** Standard Boundary conditions used for the CFD simulations

Variables	Inlet	Outlet	Wall
Velocity	Dirichlet	Homogeneous Neuman	Standard logarithmic Wall Function

Pressure	Homogeneous Neuman	Free outlet	Homogeneous Neuman

Turbulent variables	Dirichlet based on the integral lengthscale	Homogeneous Neuman	Standard Wall Function

Temperature	Dirichlet	Homogeneous Neuman	Standard Wall Function with Dirichlet or Neumann boundary conditions

A passive scalar is used to qualitatively follow particles [12]. Additional post-processing is carried out using trajectories to predict high particle density locations. In the case of passive scalar transport, the source (discretized surfaces) is considered as a Dirichlet condition. For post-processing with particles that follow the streamlines, a given number of particles are emitted from the source.

No deposition and re-suspension is taken into account at the wall with both methods.

The mesh consists of about 300,000 hexahedral cells, which represents a very high quality for such CFD computations. No grid sensitivity study has been carried out. Using our experience from previous CFD computations, the present refinement is *a priori *not sufficient for a perfect convergence of the results but appropriate to have good qualitative results. The computational domain covers the central treatment room and the two anterooms including equipment, with eight staff members and one patient located in the treatment room. Thermal sources in the simulation included 70 W for the patient considered at rest, 135 W for each of the standing and active eight staff members, 200 W for both ceiling lamps, and 965 W for the bedpan washer in the exit anteroom. Both the steady-state condition (RANS), when all doors are closed, and the transient regime (URANS), with either the entry or exit anteroom room door open, were simulated to track transportation of airborne contaminates.

### Prototype burn unit

A prototype burn treatment unit, which closely followed the initial room design used for CFD simulations, was constructed at St-Louis Hospital, Paris, France. Due to architectural constraints, the actual anterooms were slightly smaller and three exhaust grilles located at 0.15, 1.0 and 1.8 meters above the floor in the corners of the central treatment room were used instead of two. All other air supply and exhaust configurations corresponded to those of the initial room design. Supply air was treated by state-of-the-art HEPA-MD units, supplied by the company AirInSpace S.A.S., Montigny-le-Bretonneux, France, which were installed in the air distribution ventilation system. These units ensured HEPA-grade filtration and biological decontamination of the air supplied to the central treatment room. The air-flow, room pressures and temperatures were all maintained in accordance with the initial room design criteria.

The central room was equipped with an operating table and suspended overhead operating lights. Six mannequins equipped with 100 W lamps were used to simulate surgical staff in the central room. 200 W and 500 W convector heaters were also placed in the room to further simulate heat fluxes arising from diverse medical equipment.

### Measurements within the prototype burn unit

Time-series measurements of airborne particle concentrations in the treatment and anterooms were used to experimentally validate the simulated predictions of how airborne particles are transported throughout the prototype burn unit. In order to match with the CFD passive scalar particulate transportation model used, a dry inert powder having a particle size range from 0.5 to 20 μm, was aerosolized in the treatment room to mimic airborne contamination [12] The powder was injected into the air using a dust generator (SAG410, Topas) at different locations so as to trace particle movements in various areas throughout the rooms. Measurements of the airborne particle concentration were made using a 6-channel light-scattering particle counter (Climet, CI-450t) with readings covering the particulate sizes of the dry inert powder.

## Results

### CFD simulations

**Figure 2:**
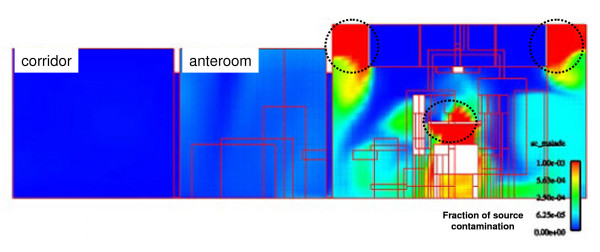
**CFD simulations**. CFD snap-shot of the treatment room, anteroom and corridor under standard operating conditions with source contamination arising from the patient table. Colors represent the fraction of source contamination, with highest concentrations in red (10^-3^) and lowest in blue (0.0). Dotted-line circles indicate zones of high airborne contamination.

The first series of simulations performed were designed to investigate the transport of patient-generated contamination in a fully staffed and equipped treatment room under realistic conditions. A CFD snap-shot from these simulations, corresponding to steady-state operation of the treatment room with continuous generation of airborne particulate matter from the patient body surface in the center of the room, is portrayed in Figure 2. The colors in the figure represent the fraction of source contamination, with highest concentrations in red (10^-3^) and lowest in blue (0.0). High concentration "hot spots" are identified in the figure by the dotted-line circles inserted over the patient's bed (source) and in the upper most portion of the room (accumulation). The figure also demonstrates that the contamination is well contained in the treatment room with only very slight amounts migrating into the anteroom and no detectable contamination in the corridor. Once the source contamination was turned off, 99% of the room contamination was evacuated within one minute. However, it is important to note that a small portion of the contamination entrained by heat convection currents into the upper part of the room between the air diffusion panels and room walls, lingered throughout the entire duration of the simulation. The dynamic representation of air-flows in the patient room shows that this accumulation was maintained by circulation eddies and that the curtain of the ceiling plenum prevented re-entry of contaminates into the patient zone (Additional file 1)

**Figure 3:**
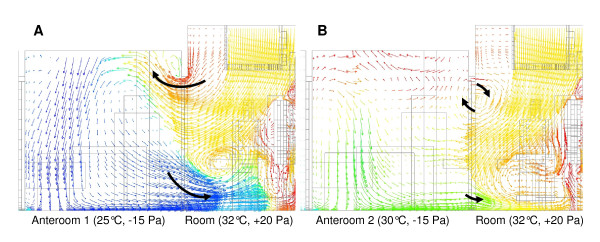
**CFD simulation of thermal convective flows**. CFD images demonstrating the thermal convective flows between rooms that have different temperatures. Arrows indicate direction and the relative air speed by their length. Colors correspond to temperature, red being the highest and blue the lowest. Large dark arrows are superimposed on the flow field lines to help visualize the overall air flows. Figure **3a **simulates a temperature difference of 7°C while **3b **one of 2°C.

Additional simulations were carried out to understand the transport of airborne contamination when the sliding door between the treatment room and an anteroom is opened. Two important cases are demonstrated in Figure 3, corresponding to imposing a large and small temperature difference between the treatment room and the anterooms. In Figure 3a the treatment room is maintained at 32°C while the anteroom is at 25°C, (ΔT = 7°C) while for Figure 3b the anteroom is elevated to 30°C, (ΔT = 2°C). The simulations clearly predict a strong influence of the temperature gradient between the rooms. Large temperature differences (*e.g. *7°C) create thermal convection, which expels warm air into the adjacent anteroom near the top of the door entrance, while cooler air from the anteroom is drawn into the treatment room at floor level. When the temperature gradient is reduced to 2°C under the same air flow conditions, the thermal convection effects become negligible (Figure 3b). The full CFD simulation of the entry sequence through the anteroom and into the patient room is shown on Movie 2 (Additional file 2). A mild convection effect is observed during access from the corridor to the entry anteroom due to the 2°C temperature difference between the two rooms. However, this only resulted in a negligible transfer of airborne contaminates. Subsequently, an intense convective effect is witnessed upon entry into the patient room, resulting in an important exchange of contaminates between the two rooms. In the exit sequence (Additional file 3) the thermal convection effect between the patient room and the anteroom was limited and resulted in a mild and transient contamination of the exit anteroom. As for the entry sequence, a mild thermal convection effect was observed between the anteroom and the corridor, but had no consequence on the corridor contamination.

### Qualitative validation of CFD findings in the prototype room

Experimental measurements were carried out to evaluate the CFD predictions and determine if the effects of heat convection currents and thermal transport influence airborne particulate contamination. For these studies the constructed treatment room was used with the ventilation system, mannequins, lights and artificial equipment all in operation so as to simulate CFD conditions and to provide a realistic model scenario.

**Figure 4:**
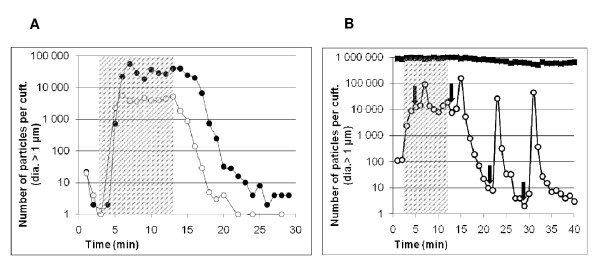
**Experimental validation of CFD findings**. Sequential particle measurement at 1 min interval during and after spiking with inert particles (dashed area). **4a**: spiking the operating table, showing accumulation of particle in the upper part of the room (30 cm from the ceiling, black circles) compared to the lower part (30 cm from the floor, open circles). **4b**: spiking the entry anteroom, showing transfer of particles from the upper part of the entry anteroom (black squares) towards the lower part of the room (open circles) when the door is opened (arrows).

To test for overhead accumulation of contaminate as indicated by the simulation results in Figure 2, the aerosol generator was placed on top of the patient's table and airborne concentration levels of particles greater than 1 μm were monitored at 30 cm from the floor and 30 cm from the ceiling, both just outside of the perimeter of the overhead air-distribution system. Simultaneous measurements were taken at one-minute intervals throughout the contamination period and after the aerosol generator was turned off. Figure 4a provides a time-series plot that summarizes the results for particles in the 1 μm-5 μm size range. The contamination period lasted approximately ten minutes and is indicated by the hatched lines in the figure. During this period a steady rise in airborne particle levels is observed which reaches a high plateau level after three minutes. Once the generator is turned off, the airborne particle concentrations follow a 4-log decrease in ten minutes. Throughout the entire period the upper-room levels exceed the lower levels by approximately one log.

To study the thermal convection of particles between rooms, the constructed burn unit was placed in operation and the airborne particle levels were monitored while the sliding room access doors were opened and closed (Figure 4b). Figure 4b presents the results for 1 μm-5 μm particle levels near the door in the upper portion of the entry anteroom at 25°C (-15 Pa) (filled squares) and in the lower portion of the treatment room at 32°C (+20 Pa) (open circles). The particle generator was placed in the treatment room and ran continuously for ten minutes (hatched area). Concentration measurements were taken simultaneously at one-minute intervals. Arrows in the figure indicate the times at which the sliding door was opened for a 10 second period. It can clearly be seen that a spike in the airborne contamination level inside the treatment room is witnessed after each door opening and that the relative importance of this spike is increased when the overall contamination is lower. In contrast to this, comparing door openings between the anteroom at 25°C (-15 Pa) and corridor at 27°C (0 Pa) with particle contamination taking place inside the anteroom, very little contaminate transfer is witnessed during the contamination period with no detectable transfer afterwards (data not shown). Noteworthy, similar trends of concentration ratios were observed for smaller particle sizes (0.5 μm-1 μm) but with lower quantitative impact of the artificial contamination period than the data presented (data not shown). Concentrations of particles for sizes above 5 μm were negligible even during the artificial contamination period (data not shown).

## Discussion

CFD is a powerful analytical tool for understanding flows and transport of microorganisms in protected environments and hospital settings [13,14]. It has already been used for improving the control of airborne particles in operating theatres showing that room geometry, airflow rates, air velocity profiles, distribution of heat sources and operating lights were determinant for the containment of infection [15,16]. However, to our knowledge, CFD has been rarely used for assisting the design of burn units [17]. Moreover, the majority of work has focused on pressure-driven air flows, and not heat-induced motion and its consequences. This later point is particularly relevant to burn units due to their unique environmental conditions and high risk of patient infection [1,4], which has served as the stimulus for us to use CFD analysis in the design of our prototype burn unit and to investigate several scenarios. To that end we have found that temperature gradients can play an important role in the distribution of airborne contaminates and careful analysis of air flows should take these into account. As seen in CFD simulations and particle tracer experiments, heat convective currents can trap suspended particles in so-called "hot spots". In itself the presence of these spots represent a contaminate source risk, and in addition to this the problem can be aggravated, leading to further risk through transport to adjacent rooms due to differential room temperatures that create thermal convective flows. This finding led us to take corrective engineering measures that called for enhanced exhaust flows in the upper section of the treatment room near the access doors.

Indeed, the risk of occasional small back flow created when doors are open simultaneously or when there is a high difference of temperature across an open door has already been considered in the design of ventilation system in healthcare facilities [18]. Here we provide a CFD evidence of this phenomenon and show its consequences in terms of real particle transfer between pressurized rooms for small particle sizes (<5 μm). We note that, due to the lack of sufficient concentrations of particles above 5 μm in our experiments, we are unable to make conclusions within this size range.

We have found that when a temperature difference of 7°C existed between the rooms, inter-room thermal convective flows predicted by CFD and qualitatively verified experimentally were important enough to transport airborne contamination into the positive pressure treatment room when the access door was opened, despite an initial 35 Pa pressure difference between the rooms when the doors are closed. However, with only a 2°C temperature difference we observed only negligible effects even when lower initial pressure differences were opposing the thermal convection (15 Pa).

These observations are consistent with recent work by Dong *et al. *[19], who report a similar phenomenon using tracer gas measurements to confirm their predictions and assumed that particle transport could ensue. The present CFD predictions are complimentary to those of Dong *et al. *and our experimental observations performed with 0.5-20 microns size particles support that transport of bacteria and fungal spores can arise from these convective flows.

## Conclusions

In conclusion, CFD simulations proved to be particularly useful for the design and optimization of the burn unit treatment room constructed at St-Louis Hospital. Our results showed the importance of airborne particle transport via thermal convection flows generated by thermal differences between rooms. Such phenomenon has potential consequences in terms of prevention of infections since we showed experimentally that particles with sizes in the range of bacteria or fungal spores were carried by the thermal flow and could bypass the protective overpressure in the patient room. Based on these data, the design of the ventilation system, the choice of room temperatures was amended and health care workers practices were adapted to prevent particle carryover. Although CFD simulation of particle transport has some limitations as a surrogate for microbial behaviour in the environment, we estimate that such phenomenon and pressure bypass is likely to occur in any pressurized room, ward or laboratory and should be considered as a risk of microbial airborne transmission.

## Competing interests

The authors declare that they have no competing interests.

## Authors' contributions

CB conducted and coordinated the CFD study and drafted the manuscript. NL conceived and carried out the experimental work. He substantially contributed to the interpretation of data and drafting of the manuscript. FC and JL carried out the experimental work at the technical level. JLR carried out the CFD study. SB contributed to CFD analysis. MC and MM conceived the draft of the pilot room and significantly contributed to data analysis and interpretation. JPL and MB conceived and supported the study at the hospital level and participated to its design and coordination. VB: substantially contributed to CFD and experimental conception, data analysis and interpretation and critical revision of the manuscript. FD conceived, conducted and coordinated the study, and drafted the manuscript. All authors read and approved the final version of the manuscript.

## Pre-publication history

The pre-publication history for this paper can be accessed here:

http://www.biomedcentral.com/1471-2334/11/58/prepub

## Supplementary Material

Additional file 1 **Movie1 - patient room**. Dynamic representation of air-flows in the patient room with source contamination arising from the patient table. Colors represent the fraction of source contamination, with highest concentrations in red and lowest in blue. A contamination accumulation is entrained by heat convection currents into the upper part of the room between the air diffusion panels and room walls. (mpeg2 movie sequence) Click here for file

Additional file 2 **Movie2 - entry sequence**. CFD simulation of the sequential entry into the patient room through the anteroom, showing a "thermal switch" between patient room and anteroom. When the door is open, (T = 1065s-1070s), warm air (32°C) is aspirated into the adjacent anteroom near the top of the door entrance, while cooler air (25°C) from the anteroom is drawn into the patient room at floor level. (mpeg2 movie sequence) Click here for file

Additional file 3 **Movie3 - exit sequence**. CFD simulation of the sequential entry into the patient room through the anteroom When the door is open, (T = 1670s-1675s) no significant transfer is observed between patient room and anteroom that were at the same temperature (32°C). (mpeg2 movie sequence) Click here for file
